# Supplementary material for: Efficacy and Safety of Neoadjuvant Gemcitabine Plus Nab-Paclitaxel in Borderline Resectable and Locally Advanced Pancreatic Cancer—A Systematic Review and Meta-Analysis
Source: Cancers (Basel). 2021 Aug 27;13(17):4326. doi: 10.3390/cancers13174326 (PMC8430874; doi:10.3390/cancers13174326)
Supplement: Supplementary file 1 [file cancers-13-04326-s001.zip › cancers-1273344-supplementary.pdf]

*Supplementary Materials*

# Efficacy and Safety of Neoadjuvant Gemcitabine plus Nab-Paclitaxel in Borderline Resectable and Locally Advanced Pancreatic Cancer–a Systematic Review and Meta-Analysis

**Marko Damm, Ljupcho Efremov, Benedikt Birnbach, Gretel Terrero, Jörg Kleeff, Rafael Mikolajczyk, Jonas Rosendahl, Patrick Michl and Sebastian Krug**

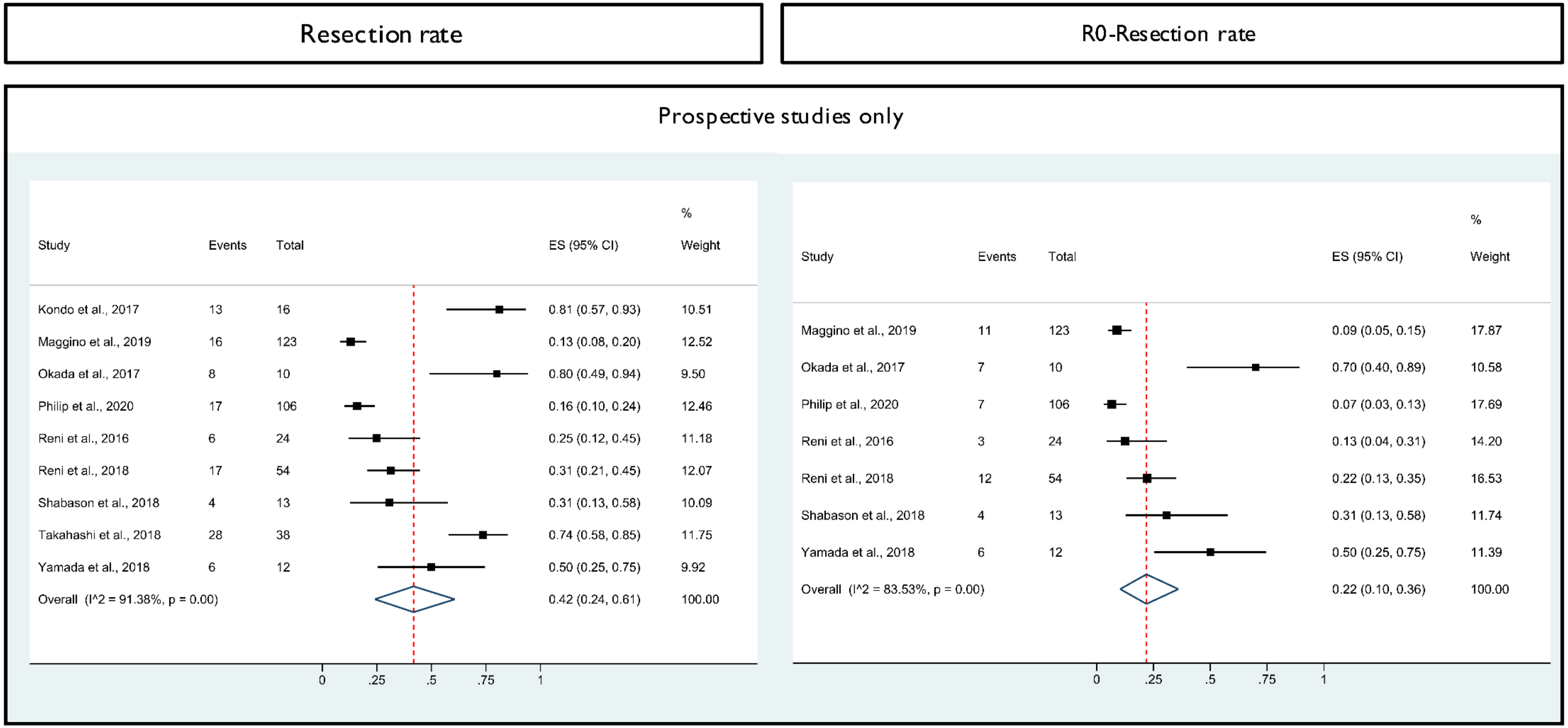


**Figure S1.** Forest plots showing the pooled proportions of resections and R0 resections (defined as the absence of tumor at the margin or a minimum distance between tumor and margin of >1 mm) of prospective studies (*n* = 396, [31,33,36,38–42,46]). Due to heterogeneity among studies, a random effects model was used. The proportions of the R0 resections were calculated on the basis of the total number of patients treated with neoadjuvant gemcitabine and nab-paclitaxel in the intention-to-treat population. The red dotted line and blue diamond shape indicate the overall pooled proportions (resection rate or R0 resection rate) including overall 95% confidence intervals (95% CI). The individual proportions of each study including 95% CI are shown in the column "ES (95% CI)". The graphical representation corresponds to the black squares and lines whereby the size of the squares reflects the respective weighting in the analysis. Abbreviation: ES, effect size.

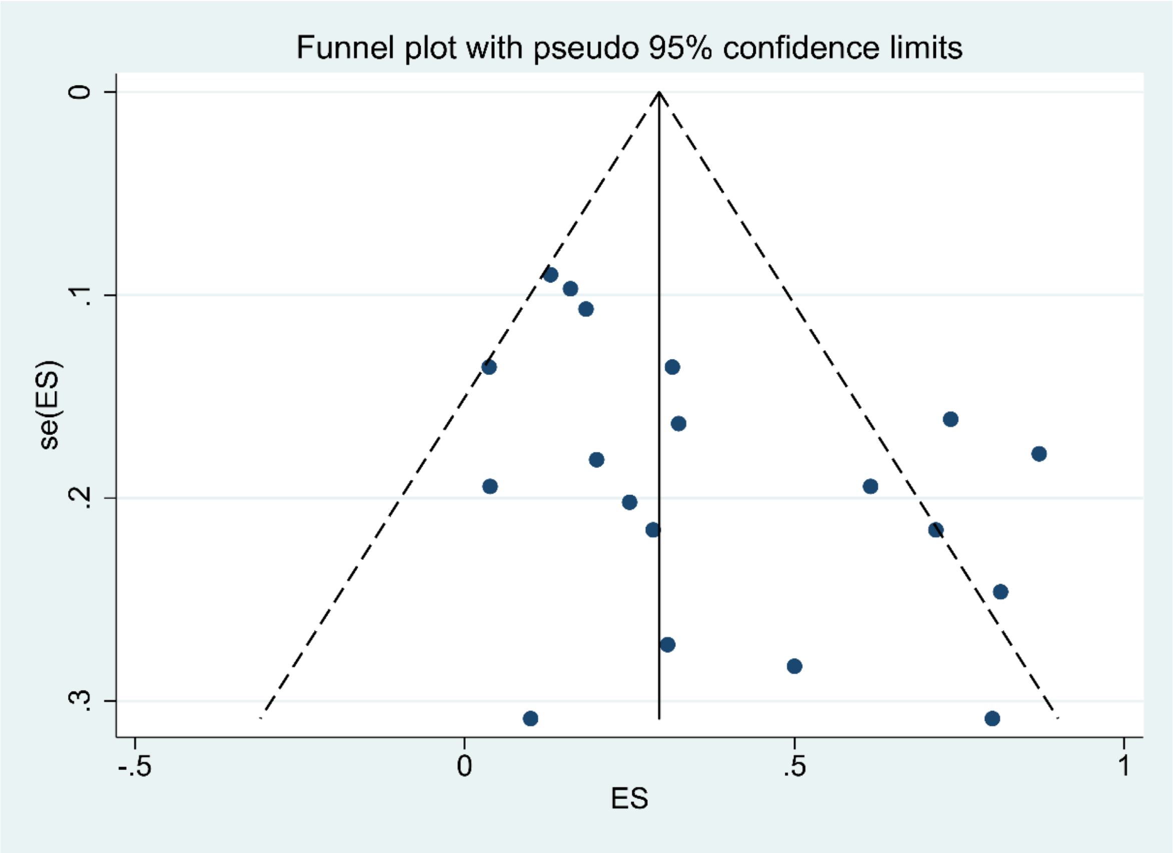


**Figure S2.** Funnel plot of all studies included in the meta-analysis ($n$ = 19, [26,28–31,33–46]). Circles represent included studies. The plot displays the results (x-axis) and the precision of the studies (y-axis). The line in the center indicates the summary proportion. The two dotted lines represent the 95% confidence limits. Symmetry about the pooled proportion line is suggestive of absence of publication bias. Abbreviations: ES, effect size; se(ES), standard error of effect size.

**Table S1.** Grade ≥ 3 adverse events of included studies [26–46].

| No. | Study | *n* | Neutropenia | | Febrile Neutropenia | | Anemia | | Thrombocytopenia | | Infection | | Neuropathy | | Death | |
|---|---|---|---|---|---|---|---|---|---|---|---|---|---|---|---|---|
| | | | *n* | % | *n* | % | *n* | % | *n* | % | *n* | % | *n* | % | *n* | % |
| 1 | Chapman et al., 2018 | 37 | 5 | 14 | NR | NR | NR | NR | 2 | 5 | 0 | 0 | 1 | 3 | NR | NR |
| 2 | Dhir et al., 2018 | 120 | NR | NR | NR | NR | NR | NR | NR | NR | NR | NR | NR | NR | NR | NR |
| 3 | Gemenetzis et al., 2019 | 87 | NR | NR | NR | NR | NR | NR | NR | NR | NR | NR | NR | NR | NR | NR |
| 4 | Gulhati et al., 2019 | 54 | NR | 12 | NR | 3 | NR | 1 | NR | NR | NR | NR | NR | 1 | NR | NR |
| 5 | Ielpo et al., 2017 *1 | 45 | 4 | 9 | NR | NR | 0 | 0 | 2 | 4 | 6 | 13 | 0 | 0 | 0 | 0 |
| 6 | Kondo et al., 2017 | 16 | 3 | 19 | 0 | 0 | 0 | 0 | 0 | 0 | 1 | 6 | 0 | 0 | 0 | 0 |
| 7 | Macedo et al., 2019 | 91 | NR | NR | NR | NR | NR | NR | NR | NR | NR | NR | NR | NR | NR | NR |
| 8 | Maggino et al., 2019 | 123 | NR | NR | NR | NR | NR | NR | NR | NR | NR | NR | NR | NR | NR | NR |
| 9 | Miyasaka et al., 2019 | 31 | NR | NR | NR | NR | NR | NR | NR | NR | NR | NR | NR | NR | NR | NR |
| 10 | Napolitano et al., 2019 | 21 | 1 | 5 | NR | NR | 1 | 5 | 1 | 5 | NR | NR | 1 | 5 | NR | NR |
| 11 | Okada et al., 2017 | 10 | 9 | 90 | 0 | 0 | 1 | 10 | 2 | 20 | NR | NR | 1 | 10 | 0 | 0 |
| 12 | Peterson et al., 2018 | 26 | NR | NR | NR | NR | NR | NR | NR | NR | NR | NR | NR | NR | NR | NR |
| 13 | Philip et al., 2020 | 106 | 35 | 33 | 4 | 4 | 12 | 11 | 4 | 4 | NR | NR | 5 | 5 | 0 | 0 |
| 14 | Reni et al., 2016 | 24 | 13 | 54 | 1 | 4 | 3 | 13 | 0 | 0 | NR | NR | 0 | 0 | 0 | 0 |
| 15 | Reni et al., 2018 | 54 | 38 | 70 | NR | NR | 6 | 11 | 3 | 6 | 6 | 11 | 5 | 9 | 0 | 0 |
| 16 | Shabason et al., 2018 *2 | 9 | 1 | 11 | NR | NR | 1 | 11 | NR | NR | NR | NR | 2 | 22 | NR | NR |
| 17 | Takahashi et al., 2018 *3 | 30 | 23 | 77 | 1 | 3 | 1 | 3 | 1 | 3 | NR | NR | 1 | 3 | 0 | 0 |
| 18 | Templeton et al., 2020 | 10 | 3 | 30 | 2 | 20 | NR | NR | NR | NR | NR | NR | 1 | 10 | NR | NR |
| 19 | Tsujimoto et al., 2019 | 30 | 22 | 73 | 0 | 0 | 2 | 7 | NR | NR | 1 | 3 | 0 | 0 | 0 | 0 |
| 20 | Weniger et al., 2020 | 21 | NR | NR | NR | NR | NR | NR | NR | NR | NR | NR | NR | NR | NR | NR |
| 21 | Yamada et al., 2018 | 12 | 7 | 58 | 0 | 0 | 0 | 0 | 1 | 8 | NR | NR | 0 | 0 | 0 | 0 |

*1 No separate results on toxicity for borderline resectable pancreatic cancer (includes resectable patients, $n = 19$). *2 Four patients were excluded because of progress after induction therapy. *3 Eight patients were excluded because of toxicity of the induction therapy. Abbreviations: NR = not reported.

**Table S2.** Quality assessment of retrospective cohort studies using the Newcastle-Ottawa Scale (NOS).

| No | Study | Selection 1 | Selection 2 | Selection 3 | Selection 4 | Comparability | Outcome 1 | Outcome 2 | Outcome 3 | NOS (Overall) | Quality * |
|---|---|---|---|---|---|---|---|---|---|---|---|
| 1 | Chapman et al., 2018 [26] | 1 | 1 | 1 | 1 | 2 | 1 | 1 | 0 | 8 | good |
| 2 | Dhir et al., 2018 [27] | 1 | 1 | 1 | 1 | 2 | 1 | 1 | 1 | 9 | good |
| 3 | Gemenetzis et al., 2019 [28] | 1 | 1 | 1 | 1 | 1 | 1 | 1 | 1 | 8 | moderate |
| 4 | Gulhati et al., 2019 [29] | 1 | 1 | 1 | 1 | 1 | 1 | 1 | 0 | 7 | moderate |
| 5 | Ielpo et al., 2017 [30] | 1 | 1 | 1 | 1 | 1 | 1 | 1 | 0 | 7 | moderate |
| 6 | Macedo et al., 2019 [32] | 1 | 1 | 1 | 1 | 2 | 1 | 1 | 1 | 9 | good |
| 7 | Maggino et al., 2019 [33] | 1 | 1 | 1 | 1 | 2 | 1 | 1 | 1 | 9 | good |
| 8 | Miyasaka et al., 2019 [34] | 1 | 1 | 1 | 1 | 1 | 1 | 1 | 0 | 7 | moderate |
| 9 | Napolitano et al., 2019 [35] | 1 | 1 | 1 | 1 | 1 | 1 | 1 | 0 | 7 | moderate |
| 10 | Peterson et al., 2018 [37] | 1 | 1 | 1 | 1 | 1 | 1 | 1 | 0 | 7 | moderate |
| 11 | Templeton et al., 2020 [43] | 1 | 1 | 1 | 1 | 0 | 1 | 1 | 0 | 6 | poor |
| 12 | Tsujimoto et al., 2019 [44] | 1 | 1 | 1 | 1 | 1 | 1 | 1 | 0 | 7 | moderate |
| 13 | Weniger et al., 2020 [45] | 1 | 1 | 1 | 1 | 1 | 1 | 1 | 0 | 7 | moderate |

*A study was scored as good quality if at least 3 points for selection, 1 for comparability and 2 for outcome were achieved. Moderate quality was defined as 2 points for selection, 1 point for comparability or 1 point for outcome. Studies of poor quality reached 1 point for selection, no points for comparability or 1 point in outcome.

**Table S3.** Risk of bias assessment of clinical trials using the Cochrane Collaboration's tool.

| No | Study | Random Sequence Generation (Selection Bias) | Allocation Concealment (Selection Bias) | Blinding of Participants And Personnel (Performance Bias) | Blinding Of Outcome Assessment (Detection Bias) | Incomplete Outcome Data (Attrition Bias) | Selective Reporting (Reporting Bias) | Other Bias | Overall Bias |
|---|---|---|---|---|---|---|---|---|---|
| 1 | Kondo et al., 2017 [31] | High risk | High risk | High risk | High risk | Low risk | Low risk | Unclear risk | High risk |
| 2 | Okada et al., 2017 [36] | High risk | High risk | High risk | High risk | Low risk | Low risk | Unclear risk | High risk |
| 3 | Philip et al. 2020 [38] | High risk | High risk | High risk | High risk | Low risk | Low risk | Unclear risk | High risk |
| 4 | Reni et al., 2016 [39] | High risk | High risk | High risk | High risk | Low risk | Low risk | Unclear risk | High risk |
| 5 | Reni et al., 2018 [40] | Low risk | Unclear risk | High risk | High risk | Low risk | Low risk | Unclear risk | Unclear risk |
| 6 | Shabason et al., 2018 [41] | High risk | High risk | High risk | High risk | Low risk | Low risk | Unclear risk | High risk |
| 7 | Takahashi et al., 2018 [42] | High risk | High risk | High risk | High risk | Low risk | Low risk | Unclear risk | High risk |
| 8 | Yamada et al., 2018 [46] | High risk | High risk | High risk | High risk | Low risk | Low risk | Unclear risk | High risk |

© 2021 by the authors. Licensee MDPI, Basel, Switzerland. This article is an open access article distributed under the terms and conditions of the Creative Commons Attribution (CC BY) license (http://creativecommons.org/licenses/by/4.0/).
